# Polychip‐A High‐Throughput Droplet Microfluidics Platform for Interrogating Microbial Interactions

**DOI:** 10.1002/advs.202523854

**Published:** 2026-07-27

**Authors:** Jeong Jae Han, Adrian Ryan Guzman, Aifen Zhou, Han Zhang, Rohit Gupte, Haemin Jung, Kaylee Delgado, Sini Skariah, Ali Sultan, Arul Jayaraman, Paul de Figueiredo, Arum Han

**Affiliations:** ^1^ Department of Multidisciplinary Engineering Texas A&M University College Station Texas USA; ^2^ Department of Electrical and Computer Engineering Texas A&M University College Station USA; ^3^ Department of Chemical Engineering Texas A&M University College Station USA; ^4^ Department of Biomedical Engineering Texas A&M University College Station USA; ^5^ Department of Microbial Pathogenesis and Immunology Texas A&M University College Station USA; ^6^ Department of Microbiology and Immunology Weill Cornell Medicine – Qatar Cornell University Education City Doha Qatar; ^7^ Christopher S. Bond Life Sciences Center University of Missouri Columbia USA; ^8^ Department of Molecular Microbiology & Immunology University of Missouri School of Medicine Columbia USA; ^9^ Department of Veterinary Pathobiology University of Missouri Columbia USA; ^10^ Department of Chemical and Biomedical Engineering University of Missouri Columbia USA

**Keywords:** antimicrobial discovery, droplet microfluidics, environmental microorganisms, high‐throughput screening, polymicrobial interactions

## Abstract

Microbial interactions are fundamental to ecology, health, and disease, yet high‐throughput tools to study these complex relationships remain scarce. We introduce Polychip, a fully integrated, high‐throughput droplet microfluidics platform that revolutionizes microbial interaction screening. By seamlessly combining six microfluidic operations onto a single chip, Polychip achieves 99.7% efficiency and accelerates screening by 11–14 times compared to traditional high‐throughput liquid handling robotic methods, with minimal human input. Using the Polychip, we screened 2.24 × 10^6^ soil‐extracted microorganisms against the multidrug‐resistant (MDR) pathogen *Pseudomonas aeruginosa*. This process recovered 1.96 × 10^4^ hit droplets with an antimicrobial activity confirmation accuracy of 48%. Three environmental isolates (*Stenotrophomonas* sp.) exhibiting high potency and broad‐spectrum antimicrobial activity were identified through this screen. Supernatants from these three environmental isolates suppressed growth of both gram‐negative MDR pathogen *Acinetobacter baumannii* and gram‐positive methicillin‐resistant *Staphylococcus aureus* (MRSA). Through whole‐genome sequencing, metabolic pathway analysis, and mass spectrometry, we identified the secreted compound as enterochelin. This demonstrates Polychip's unprecedented single‐cell resolution and high throughput screening capability in rapidly discovering antimicrobial activities, opening new frontiers in combating resistant microbial pathogens, as well as more broadly advancing microbial ecology research.

## Graphical abstract

Polychip, a fully integrated droplet microfluidics platform, enables high‐throughput, single‐cell resolution screening of polymicrobial interactions. By seamlessly combining six automated microfluidics operations on a single chip, the system accelerates antimicrobial discovery by 11 to 14 times compared to traditional robotic methods. Screening millions of soil microorganisms identifies potent environmental isolates producing broad‐spectrum antimicrobial compounds against multidrug‐resistant pathogens.

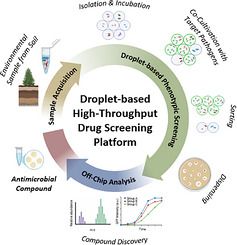

## 1 Introduction

Microorganisms live in communities that drive the global biogeochemical cycling of elements and impact the health of the hosts, including animals and plants. The interactions between community members are the foundation for maintaining the community structure and function shaped by microbial ecological and environmental interactions. Advances in metagenomics and proteomics have revealed the diversity of environmental microorganisms and molecules they produce and exchange [1, 2], as well as complex microbial interactions [3]. High‐throughput analyses of microbial interactions have broad utility, from better understanding of microbial community functions, stability, and evolution, to revolutionizing natural product discovery from environmental microbial communities. Yet scalable and high‐resolution experimental platforms capable of performing the multi‐step microbial interaction assays required for such advances remain elusive.

Most polymicrobial interaction assays require many sequential experimental steps, but traditional testing and screening methods are limited due to the lack of resolution, throughput, and high labor requirements, preventing effective investigation of complex microbial interactions requiring single‐cell behavioral analysis at scale [4, 5, 6]. Plating‐based methods are highly accurate, but labor intensive and low throughput. High‐throughput assays such as flow cytometry provide single‐cell‐resolution analysis. However, they are not suitable for polymicrobial interaction assays, as such interactions cannot be interrogated in bulk culture. Importantly, many microbial interaction assays require multiple handling steps. Whereas robotic liquid handling platforms are ideal platforms to perform complex multi‐step assays, they remain costly and thus are typically only utilized in industry settings or dedicated high‐throughput screening laboratories. Moreover, they can be difficult to program, and still only provide moderate throughput. These features make them ill‐suited for screening large environmental microbial libraries or synthetic libraries where millions of cells are typically tested [7, 8].

Several single‐cell‐resolution microfluidic platforms have been developed. For example, iChip supports the cultivation of diverse environmental microbes. However, it does not have the capacity to efficiently screen microbial interactions at scale [9]. Furthermore, this platform possesses limitations in high‐efficiency cell manipulation, single‐cell resolution analysis, precise incubation control, and seamless integration of multiple functional steps within a single platform.

Droplet microfluidics offers unparalleled opportunities to address limitations in screening complex microbial communities [10, 11]. Droplet microfluidic systems encapsulate reagents and cellular contents within a pico‐liter‐scale water‐in‐oil emulsion, producing identical droplets with varying contents, functioning as isolated pico‐liter‐volume bioreactors for high‐throughput screening [12, 13, 14, 15]. These systems significantly reduce reagent costs, enhance throughput, enable parallel analyses, and minimize footprints compared to conventional multi‐well plate‐based assays. However, for droplet microfluidic systems to successfully screen large microbial libraries, extremely low error rates in droplet handling, including generation, synchronization, merging, or sorting must be achieved to ensure overall high efficiency and accuracy [16, 17]. Given the advantages of droplet microfluidic systems, it is perhaps no surprise that several systems have been developed to interrogate microbial interactions. For example, the kChip and digital microfluidic chips have demonstrated feasibility in constructing and analyzing synthetic microbial communities [13, 18, 19]. However, these systems have failed to display high‐throughput assay capabilities and precise droplet manipulations, at scale.

Conventional droplet screening systems have also displayed limitations in managing complex workflows. For example, most droplet microfluidics‐based screening platforms incorporate only one to three key droplet manipulation steps – droplet generation, merging, and sorting – either integrated within a single microfluidic chip [14, 20] or connected via tubing [21]. While these platforms enable high‐throughput screening using droplet‐based techniques, they still require significant human intervention to ensure precision. Moreover, they lack the capability for adaptive cultivation and do not support sequential screening across multiple batches, making large‐scale microbial screening challenging. Beyond these technical limitations, there is a notable gap in demonstrating a complete end‐to‐end workflow – screening pipelines from environmental sample acquisition to isolating microorganisms that show desired phenotype of interest – within such droplet microfluidic platforms. This gap hinders the readiness of such platforms for broad applications.

We present an integrated droplet microfluidics platform named Polychip (polymicrobial interaction on a chip) that overcomes these limitations by enabling high‐throughput, single‐cell resolution, high‐efficiency, and fully automated screening of polymicrobial interactions. The fully integrated system employs multiplexed first‐in first‐out (FIFO) droplet incubation chambers with integrated microvalves to maintain uniform cultivation times across a large library of monodispersed droplets throughout the multi‐step droplet microfluidics assay, ensuring a consistent phenotypic output over the entire duration of the screening assay (tens of hours to days). By integrating recent innovations in high‐fidelity droplet synchronization before merging [22] and error‐free droplet merging [17], Polychip enables continuous operation of polymicrobial interaction assays that require six droplet manipulation steps to be performed in sequence with high precision and minimal manual intervention, thereby minimizing human error and enhancing reliability. Here, we describe how the Polychip platform was successfully deployed to screen and identify environmental microorganisms that produce antibiotics that suppress the growth of multidrug‐resistant (MDR) *Pseudomonas aeruginosa*, MDR *Acinetobacter baumannii*, and methicillin‐resistant *Staphylococcus aureus* (MRSA), thereby establishing the cogency of the technology as an antimicrobial discovery platform.

## 2 Results

### 2.1 Polychip Operating Principles

**FIGURE 1:**
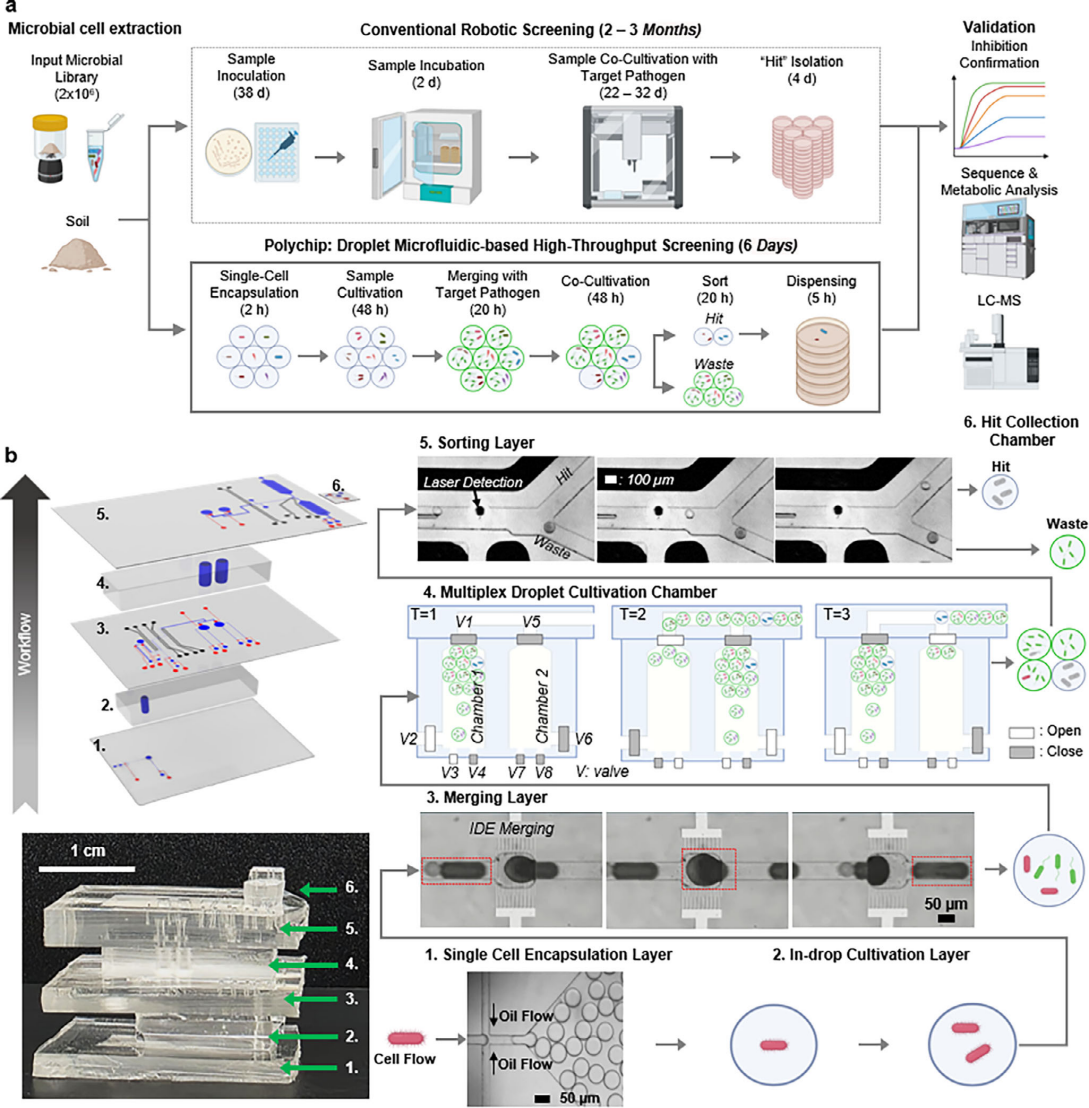
Working principle of the Polychip platform. (a) Comparison of the droplet microfluidics‐based Polychip screening workflow (bottom row) to a conventional liquid handling robot‐based screening workflow (top row), including cumulative processing time. (b) Illustration (top left) and images (bottom left) of the multi‐layer Polychip structure, along with microscopic images and illustrations that describe the key droplet manipulation steps of screening an environmental microbial library to identify and select isolates having antimicrobial activities.

The structure and workflow of the Polychip system (Figure 1) were designed to replicate key stages of a conventional liquid handling robotic screening pipeline [23], including single‐cell isolation from a microbial library, incubation for clonal expansion, co‐cultivation with target pathogens, followed by sorting and recovering isolates that show antimicrobial activities. For this time‐comparison, the Polychip assay time and the robotic screening time both specifically exclude the off‐chip validation steps. First, single‐cell droplet encapsulation and in‐drop cultivation replicate sample inoculation and incubation, respectively. Second, merging of cultivated droplets with droplets containing target pathogen followed by co‐cultivation replicates co‐cultivation performed in multi‐well plates to observe inhibition. Finally, sorting of droplets based on antimicrobial activities within the droplets (based on fluorescent readout) and dispensing the “hit” droplets at single‐droplet resolution onto agar plates replicates the “hit” isolation typically performed across hundreds of agar plates.

Here, screening soil microorganisms for their antimicrobial activities at single‐cell resolution against a target pathogen was utilized as a demonstration case of the Polychip platform. In the case of a conventional workflow (Figure 1a, top row), robotic instruments could be adopted to perform the necessary assay steps such as isolation/incubation of an input microbial library and co‐cultivating them with a target pathogen on conventional agar plates or well‐plates to observe antimicrobial activities. This typically takes 2 – 3 months to screen 2×10^6^ microorganisms. The Polychip workflow (Figure 1a, bottom row) comprises three key high‐efficiency droplet microfluidic techniques, namely single‐cell droplet encapsulation and cultivation, droplet merging with pathogen‐containing droplets and co‐cultivation, and droplet detection/sorting. Completion of one round of Polychip screening was designed to take only 5 – 6 days, a 11 – 14‐fold decrease in assay time compared to conventional workflows.

The specific timeline for each discrete Polychip step used here is as follows: cell‐encapsulated droplet generation (2 h), initial in‐drop cultivation for clonal expansion (48 h), droplet merging with pathogen‐containing droplets (20 h), in‐drop co‐cultivation (48 h), droplet detection and sorting (20 h), and droplet dispensing (5 h), totaling approximately ∼6 days. This high‐throughput processing ensures that the entire droplet library can be analyzed well within the stability limits of microdroplets, as previous studies have demonstrated in‐drop cultivation exceeding 14 days [24].

The platform exploits the natural buoyancy of aqueous droplets in carrier oil, sequentially processing each droplet manipulation step from the bottom to top layer of the Polychip. First, in the bottom‐most layer (Figure 1b, Step 1), environmental microorganisms were encapsulated at single‐cell resolution into 50 µm diameter droplets (65.5 pL). Next, these droplets flowed into a vertical droplet cultivation chamber (2 mm in diameter, 10 mm in height) holding up to ∼4 million droplets, and were cultured for 48 h (Figure 1b, Step 2), long enough to accommodate the varying doubling times and metabolic activities of diverse ranges of environmental microorganisms (Figure S1). The buoyancy of droplets enabled first‐in first‐out droplet manipulation [25], ensuring that all droplets experienced the same cultivation time. After cultivation, droplets containing environmental microorganisms were flown out from the top of the droplet cultivation chamber to a droplet merging zone composed of interdigitated electrodes [17, 22], where they were merged (Figure 1b, Step 3) with droplets (85 µm in diameter, 321.5 pL) encapsulating green fluorescence protein (GFP)‐expressing target pathogens (Figure S2). These merged droplets (90 – 100 µm in diameter ≈ 450 pL) were then cultured for an additional 48 h in a multiplex droplet cultivation chamber. This structure consists of two cylindrical droplet cultivation chambers (2 mm in diameter, 10 mm in height) controlled by eight microvalves (V1 and 5: cultured droplet release, V2 and 6: oil release, V3 and 7: droplet inlet, V4 and 8: oil infusion) to enable sequential cultivation of multiple droplet sets (Figure 1b, Step 4). This enabled the Polychip platform to conduct back‐to‐back droplet screening with minimal wait time to maximize the assay throughput, enabling large microbial libraries to be screened in one experimental step (see electronic supplementary document and Figure S3 for detailed operation scheme). The growth of the target pathogen in each droplet was monitored by the GFP intensity of the merged droplet. Here, after 48 h of co‐cultivation in the multiplex droplet cultivation chamber, droplets containing environmental microbes that exhibited antimicrobial activities showed low or no GFP intensities. In contrast, droplets containing environmental microbes with no antimicrobial activities resulted in robust growth of GFP‐expressing pathogens. In the droplet sorting layer, droplets reflowing out of the multiplex droplet cultivation chambers were detected for their GFP intensities, and only those showing low or undetectable GFP intensity were collected in the sorting layer as “hits” (Figure 1b, Step 5). Droplet sorting was performed using a previously developed dielectrophoretic linear droplet sorter [26] (see electronic supplementary document and Figures S4–S9). After sorting, the “hit” droplets were transported to a “hit” droplet collection chamber (Figure 1b, Step 6) and then dispensed onto agar plates for downstream validation (Figure S10).

In terms of throughput, the droplet generation, merging, and sorting steps were operated at 300–400, 30–50, and 30–50 droplets per second, respectively. These conditions were previously optimized to achieve efficiencies of 100%, 100%, and 99.78 ± 0.19%, for droplet generation, droplet merging, and droplet sorting, respectively [17, 26, 27], resulting in a combined overall efficiency of 99.7%. Detailed information about the Polychip efficiency and the droplet‐specific assay time are provided in the supplementary text, as well as in Table S1, Figures S11 and S12.

### 2.2 Polychip Identification of Antimicrobial Activities

**FIGURE 2:**
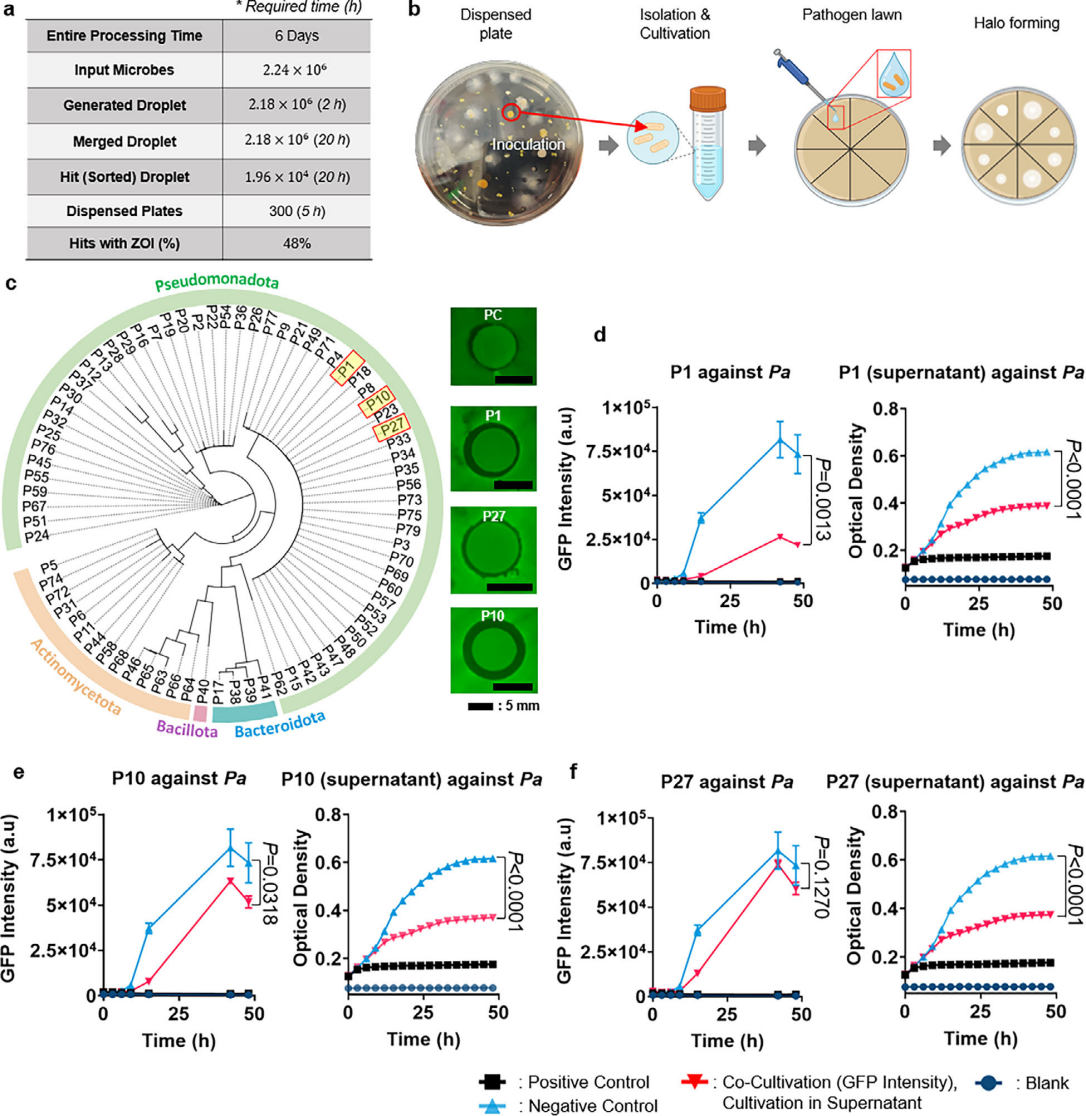
Polychip antimicrobial discovery screen of soil microbial library against MDR *P. aeruginosa*. (a) Summary of Polychip screening processes and time it took for each key step. (b) ZOI‐based antimicrobial activity assay to confirm the “hits” against MDR *P. aeruginosa*. (c) Phylogenetic tree showing diversity profiles of the 79 “hits” exhibiting confirmed antimicrobial activity. (d‐f) Off‐chip validation of the top three “high‐priority” isolates (P1, P10, and P27 based on the size of ZOIs) for their antimicrobial activities using live “hit” cell co‐culture as well as their supernatant co‐culture with MDR *P. aeruginosa* (red line), in comparison to the positive control (black line: MDR *P. aeruginosa* cultivation with 50 µg/mL of Kanamycin), the negative control (blue line: pathogen cultivation without antibiotics), and the blank (dark blue: media only). Data are presented as mean ± SD (n = 3). Statistical significance between different groups was analyzed by using Student's T‐test.

A Polychip screen was conducted to discover soil microorganisms exhibiting antimicrobial activity against GFP‐expressing MDR *P. aeruginosa* (Figure 2a). An environmental microbial community was extracted from soil located in a tall grass prairie ecosystem area (Oklahoma, USA) [28], diluted in R2A media, and then encapsulated into 50 µm diameter droplets on average one cell per droplet. Nutrition‐poor R2A was used to obtain relatively high diversity sample [24]. The individual microbial cells were cultivated in each droplet for 48 h to allow reasonable growth (growth to 50 – 500 cells per droplet) of most environmental bacteria except for very slow‐growing strains. The resulting 2.24×10^6^ droplets were then merged with MDR GFP‐*P. aeruginosa*‐containing droplets, incubated for 48 h (co‐culture), and then sorted based on the GFP intensity of droplets. The resulting 1.96×10^4^ “hit” droplets showing low GFP intensity (0.89% of the input population) were then dispensed onto solid media agar plates at single‐droplet resolution and cultivated for 48 h. A collection of 164 morphologically diverse “hit” colonies was picked for further analysis (Figure 2b). Using the Kirby‐Bauer test [29], 79 “hit” strains (48% of the 164 tested) that exhibited high antimicrobial activity with a zone of inhibition (ZOI) diameter > 8 mm were selected as “high‐priority” candidates. This 8 mm threshold was based on the ZOI of the antibiotic control (Kanamycin, 50 µg/mL). Therefore, samples that outperformed the positive control (*ZOI_hit_*  >  *ZOI*_*Pos* *Cont*_ ≈ 8 *mm*) were prioritized. The high confirmation rate (48%) of this antimicrobial activity confirmation step was achieved due to setting a stringent sorting threshold, targeting less than 1% of the total droplets based on their GFP intensity. Specifically, the intensity threshold for sorting “hit” droplets was approximately 20 – 100 (a.u.), which was 150 – 750‐fold lower than the GFP intensity of waste droplets (∼15,000 a.u.) containing 4 × 10^2^ – 4 × 10^3^ GFP‐*P. aeruginosa*. Next, the phylogenetic diversities of the top 79 “hits” were analyzed using full‐length 16S sequencing (Figure 2c). The results showed four phyla, with 60, 14, 4, and 1 “hits” associated with *Pseudomonadota*, *Actinomycetota*, *Bacteroidota*, and *Bacillota*, respectively. The top three “hits” (named P1, P10, and P27) showing the strongest antimicrobial activity against the MDR *P. aeruginosa* belonged to the *Pseudomonadota* phylum. Their antimicrobial activities were further tested by co‐incubating either live cells or culture supernatants with MDR *P. aeruginosa*. In both cases, they showed significant (*p*<0.0001) growth inhibition (P1> P10> P27) (Figure 2d‐f). The best‐performing isolate P1 demonstrated ∼70% and ∼50% reduction in MDR *P. aeruginosa* growth when using the live cell co‐culture or the supernatant co‐culture, respectively.

### 2.3 Broad Spectrum Antimicrobial Activity

**FIGURE 3:**
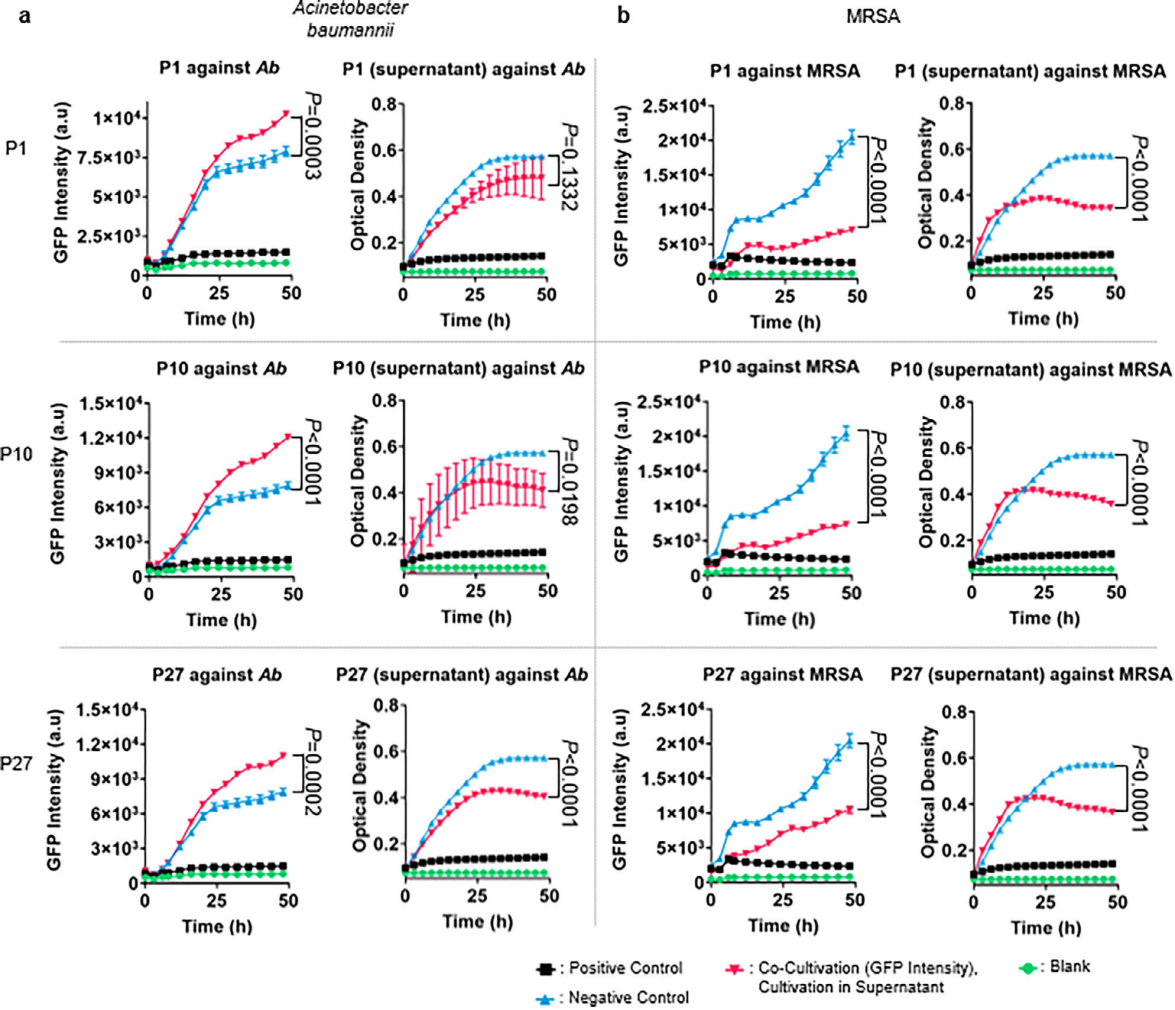
Top three “hits” P1, P10, and P27 in both live cell and extracted supernatant format co‐cultured against two additional pathogens to assess their broad‐spectrum activities. (a) MDR *A. baumannii* and (b) MRSA. Positive control (black line: target pathogen cultivation with 50 µg/mL of Kanamycin); Negative control (blue line: target pathogen cultivation without Kanamycin); Co‐cultivation (red line: “hit” cells or supernatant co‐cultivation with the target pathogen); Blank (dark blue: media only). Data are presented as the mean ± SD (n = 3). The statistical significance between different groups was analyzed by Student's t‐test.

To assess the spectrum of antimicrobial activity of the “high priority” isolates discovered through the Polychip screen, antimicrobial susceptibility tests against MDR pathogens including gram‐negative *A. baumannii* and gram‐positive MRSA were performed using P1, P10, and P27 (Figure 3). GFP signal intensities were monitored to evaluate the antimicrobial activities of live P1, P10, and P27 cells co‐cultured with GFP‐expressing pathogens, while OD_600_ readings were monitored to evaluate the antimicrobial activities of P1, P10, and P27 supernatant against target pathogens. When tested against MDR *A. baumannii*, the supernatants from P1, P10, and P27 strains suppressed the growth by approximately 16%, 28%, and 29%, respectively, compared to the negative controls (Figure 3A, right column). In the case of MRSA, both live cells and supernatants of P1, P10, and P27 exhibited significant antimicrobial activities. P1, P10, and P27 live cell co‐culture suppressed the growth of MRSA by 65%, 64%, and 49%, respectively, compared to negative controls (Figure 3B, left column), while the supernatants of P1, P10, and P27 suppressed the growth of MRSA by 40%, 37%, and 36%, respectively (Figure 3B, right column). These results demonstrate that the supernatants exhibit antimicrobial activities against both gram‐positive and gram‐negative pathogens, implying that the antimicrobial compounds in these supernatants may be broad‐spectrum antibiotics.

### 2.4 Identification of Antimicrobial Molecules

**FIGURE 4:**
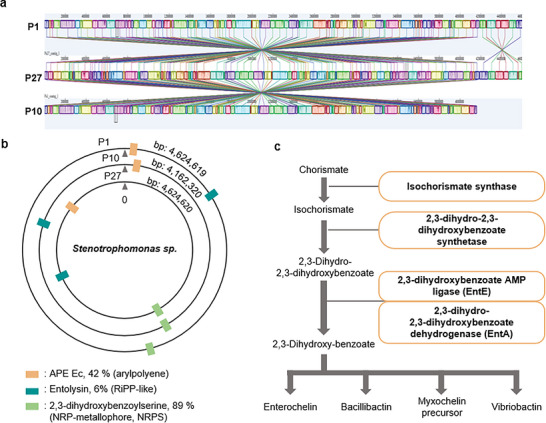
Analyses to identify the potential antimicrobial compounds produced by the three strongest “hits,” P1, P10, and P27. (a) Whole genome alignment based on MAUVE analysis of P1, P10, and P27. (b) Closest matching known cluster of secondary metabolic products analyzed by AntiSMASH. (c) Identified secondary metabolic pathways present in the genomes of P1, P10, and P27.

To identify the potential key biosynthetic gene clusters (BGCs) that contribute to the antimicrobial activities of the three most promising strains (P1, P10, and P27), whole genome sequencing and secondary metabolism pathway analyses using AntiSMASH [30] were conducted (Figure 4). The whole genome sequences showed that P1, P10, and P27 were *Stenotrophomonas* sp., a Gram‐negative, rod‐shaped bacterium known for its metabolic versatility, environmental resilience, and roles in bioremediation, plant growth promotion, and opportunistic infections [31], with genome sizes (bp) of 4 624 619, 4 162 320, and 4 624 620, respectively. Genome sequence alignment based on MAUVE analysis [32] showed that P1 and P27 are very similar (Figure 4a). The AntiSMASH results showed that all three “hits” had three secondary metabolic pathway gene clusters that are involved in the production of arylpolyene (APE), entolysin (RiPP‐like), and 2,3‐dihydroxybenzoylserine (NRP‐metallophore, NRPS), with similarities of 42%, 6%, and 89%, respectively, based on sequences in the Minimum Information about a Biosynthetic Gene cluster (MIBig) [33] database (Figure 4b). To discover which of the compounds showed antimicrobial activity against MDR *P. aeruginosa*, the NRPS with the highest similarity of 89% was chosen for secondary metabolism pathway analysis (Figure 4c). Four genes encoding enzymes catalyzing reactions from chorismate to 2,3‐Dihydroxy‐benzoate, including isochorismate synthase, 2,3‐dihydro‐2,3‐dihydroxybenzoate synthetase, 2,3‐dihydroxybenzoate AMP ligase (EntE), and 2,3‐dihydro‐2,3‐dihydroxybenzoate dehydrogenase (EntA) were identified in the genomes of the top three “hits.” Based on the pathway analysis using the Kyoto Encyclopedia of Genes and Genomes (KEGG) database [34], four compounds, including enterochelin, bacillibactin, myxochelin precursor, and vibriobactin were identified as potential siderophores, low‐molecular‐weight iron‐chelating compounds that may have contributed to the observed antimicrobial activities [35, 36]. In all, genome and gene level analysis suggested that these molecules were candidates responsible for the antimicrobial activities uncovered in our Polychip screen.

### 2.5 Confirmation of the Antimicrobial Compounds

**FIGURE 5:**
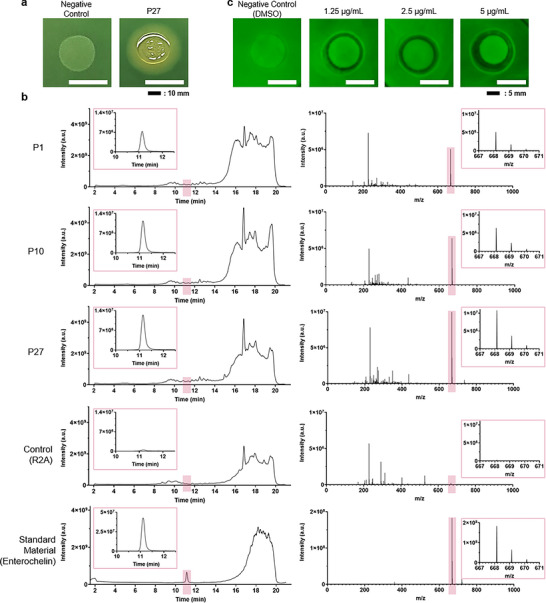
Confirmation of the discovered antimicrobial compound from strain P1, P10, and P27. (a) Siderophore detection from the supernatant using the CAS agar testing method. (b) Enterochelin detection in the supernatant using LC‐MS. (c) Kirby‐Bauer test confirming the dose‐dependent effect of enterochelin, the likely molecule produced by P1, P10, and P27.

To evaluate siderophore production induced by iron‐limited media and the antimicrobial activity of the siderophores produced by the top three hits P1, P10, and P27, we applied multiple assays, including Chrome azurol S (CAS) agar [37], LC–MS, and Kirby–Bauer tests (Figure 5). The CAS agar test for siderophore detection [37] yielded clear halos for all three “hits” under iron‐limited conditions (FeCl3, 2.7 µg/mL), as shown in Figure 5a and Figure S13, suggesting the presence of siderophores in their respective supernatants. Furthermore, through Kirby‐Bauer tests using a thin lawn of the target pathogen (MDR Pseudomonas aeruginosa) on top of R2A agar containing different concentrations of iron (FeCl3, 13.5 and 27 µg/mL), we observed that the top three “hits” showed reductions in antimicrobial activity (P1, P10, and P27) as the con‐centration of FeCl3 increased (Figure S14). These results strongly suggest that the identified antimicrobial compound contains siderophore [35, 36]. We next performed LC‐MS analyses of the extracts [36] from the supernatants to verify their production by P1, P10, and P27 (Figure 5b). The retention times (approximately 11.1 m) and mass spectrum of the unique peaks identified in the supernatant samples of P1, P10, and P27 were the same as the enterochelin standard, indicating the presence of this compound in their supernatants. Next, Kirby‐Bauer tests showed the dose‐dependent effect of enterochelin standard against MDR *P. aeruginosa*. The results also showed increased ZOI when increasing concentrations of enterochelin, from 1.25 to 5 µg/mL, were spotted onto solid media (Figure 5c). Taken together, these analyses indicate that P1, P10, and P27 indeed produce enterochelin, which exhibits antimicrobial activity against target MDR pathogen *P. aeruginosa*, *A. baumannii*, and MRSA.

## 3 Discussion

Low throughput, difficulties in reproducibility, and high error rates in discovery are typical bottlenecks that hinder natural product drug screening of environmental microbial libraries. Although liquid handling robot‐based screening technologies have significantly reduced human labor, many of these bottlenecks persist. The microfluidic Polychip (polymicrobial interaction assay chip) platform described here was designed to overcome these hurdles by enabling compartmentalization of individual environmental microorganisms into pico‐liter‐volume water‐in‐oil emulsion droplets, allowing clonal expansion of individual isolates within these droplets, then adding target microorganisms of interest into each droplet and co‐cultivating them, followed by sorting droplets that exhibit activities against the target microorganism, and finally retrieving the environmental isolates for further analyses. In this work, antimicrobial activity discovery was used as an example case for demonstrating the utility of the high‐throughput, single‐cell‐resolution Polychip.

As an example, Polychip screen of 2.18×10^6^ droplets encapsulating soil microorganisms against MDR *P. aeruginosa* identified, within 6 days, a small number of droplets that exhibited potential antimicrobial activities (<1% of input library, 1.96×10^4^ “hit” droplets). A total of 164 isolates were picked for confirmation based on morphological differences, of which ∼48% were confirmed to show strong antimicrobial activities, demonstrating the high selectivity of the Polychip screening system. Importantly, the Polychip‐based screening could be conducted 11 – 14‐fold faster than conventional liquid handling robotics‐based methods. The reproducibility of the Polychip system was supported by conducting an additional screen against MDR *A. baumannii* (Table S2), where screening 3.14 × 10^6^ environmental microorganisms resulted in 3.05 × 10^6^ cell‐encapsulated droplets, of which 2.77 × 10^4^ droplets were sorted as “hit droplets”. A total of 78 isolates were picked for confirmation, resulting in 24 isolates (31% of the isolates tested) confirmed to show strong antimicrobial activities.

This outcome was enabled by the fact that the Polychip system integrates all droplet microfluidics functions into a single chip format to maximize system efficiency while minimizing manual handling steps that can increase error. Typically, most droplet microfluidic systems enable only 1–3 droplet manipulation steps on a single chip due to the difficulties in integrating more than a few steps into a single chip. Thus, if more complex droplet microfluidics procedures are needed, multiple functionally distinct chips are often connected with tubing [21]. However, this concatenation of devices inevitably necessitates complex manual handling steps, leading to unwanted droplet shearing, merging, or shrinkage as droplets transition from one chip to another during the droplet microfluidics assay steps. The developed Polychip platform integrates all essential droplet assay manipulation and screening components (single‐cell encapsulation droplet generator, a bottom‐up FIFO droplet cultivation chamber, high‐efficiency droplet merger, multiplexed droplet cultivation chambers with valving controls for automation, and droplet sorter) into a single chip format. In addition to enhancing the efficiency and reliability of the overall workflow, the integrated design simplifies and enhances the user experience through a field programmable gate array (FPGA)‐based graphic user interface (GUI) that automates all droplet microfluidic handling steps (Figures S5–S9). Table 1 provides a comparative analysis of the PolyChip platform against robotic‐arm‐based screening or conventional microfluidic screening methods.

**TABLE 1:** Comparison of the performance of Polychip to conventional liquid handling robot, iCHIP, and kCHIP.

Key Metrics	Conventional Liquid Handling Robot	iCHIP	kCHIP	Polychip (this work)
Single Run Capacity	10^3^ – 10^4^ Samples	∼ 384 Isolates	∼ 10^5^ Droplets	∼ 4 × 10^6^ Droplets
Operational Accuracy	>99% (Manual)	Variable (Assembly Dependent)	∼ 90 – 95%	∼ 99.7%
Single Cell Assessment	No	No	No	Yes
Required Volume	Microliter (>1 µl)	25 – 80 µl	10 – 20 nL	65 pL
Integrated Steps	0 (Discrete Steps)	1 (Cultivation)	2 (Generation/Merging)	5 (Generation/Merging/ Sorting/Incubating)
Processing Time^a^ (to screen 2 × 10^6^ cells)	2 – 3 Months	Low Throughput	∼ 15 – 20 Days	5 – 6 Days
Integrated Cultivation System	No	No	No	Yes (On‐Chip FIFO)

^a^ Processing time: The time required to complete the screening of 2 × 10^6^ cells to obtain candidates.

The application of this Polychip platform for soil microorganism screening against MDR *P. aeruginosa* for antimicrobial natural product discovery resulted in 164 potential “hits.” These “hits” underwent phenotypic confirmation analysis where approximately half of the “hits” exhibited strong antimicrobial activity. The three most promising “hits” based on the size of ZOI were further analyzed, confirming that both the live “hit” cells and their supernatants exhibited growth inhibition against MDR *P. aeruginosa*, reducing pathogen growth by ∼70% and ∼50%, respectively. Using a simple mathematical estimation based on this result, Polychip could theoretically generate up to 300 000 antimicrobial “hits” within three months (300 000 ≈ 1.96×10^4^×15 = [number of “hit” droplets per 6 days]×[15 folds]), offering unprecedented scalability for the discovery of antimicrobial compounds.

To identify the mechanism underlying the antimicrobial activity of the three most potent “hits” (P1, P10, and P27), multiple gene‐level analyses were conducted, including whole‐genome sequencing, AntiSMASH analysis, and secondary metabolism pathway analysis. An additional Phenotypic confirmation of the most potent “hits” (P1, P10, and P27) against broad spectrum of pathogen panel consisting of MDR *A. baumannii* (gram‐negative) and MRSA (gram‐positive) was conducted, where both living cells and the supernatants of the most potent “hits” suppressed the growth of the target pathogens (Figure 3). To identify the compound responsible for antimicrobial activities, CAS agar, LC‐MS, and Kirby‐Bauer tests were conducted. Based on the aforementioned methods, we identified enterochelin as the compound that resulted in antimicrobial activity. This finding supports the potential of Polychip as a promising alternative to conventional antimicrobial screening methods, as its true strength lies in enabling massively parallel, multi‐step operations for biological applications such as drug discovery and microbial screening, where ultra‐high efficiency and accuracy are essential.

Enterochelin (enterobactin) is a catecholate siderophore that exhibits antibacterial activity primarily through iron sequestration, which can inhibit the growth of certain bacteria. Its complexities with specific metals can directly inhibit bacterial growth by interfering with iron uptake [38, 39]. Moreover, enterochelin can serve as a natural vector for delivering antibiotic molecules into Gram‐negative bacteria, significantly enhancing the efficacy of conjugated antibiotics by hijacking bacterial iron transport systems [40]. Synthetic conjugates of enterochelin linked to β‐lactam antibiotics (e.g., ampicillin or amoxicillin) have been developed to exploit the siderophore's iron uptake pathway to deliver antibiotics more effectively into gram‐negative bacteria such as *Escherichia coli*. These conjugates show significantly enhanced antibacterial activity (up to 1000‐fold reduction in minimum inhibitory concentration) compared to the parent antibiotics, particularly under iron‐limited conditions [41]. Therefore, Polychip successfully resolved antimicrobial activities of potential clinical relevance.

By addressing key bottlenecks in traditional droplet‐based screening approaches, the Polychip platform paves the way for next‐generation high‐throughput screening that can be seamlessly integrated into a wide variety of applications. Although this proof‐of‐concept study utilized GFP‐expressing target pathogens, Polychip is also compatible with not only screening other fluorescence‐expressing high‐priority pathogens as well as conducting screening against non‐engineered target pathogens. For example, fluorescent labeling [42, 43, 44] of target pathogens is an option for screening clinically or environmentally relevant target organisms. Additionally, the Polychip also supports conducting the screening under anaerobic conditions by placing the system within an anaerobic chamber since the chip is made of gas‐permeable PDMS. Because the platform is managed through a GUI on a connected laptop, users can operate the system and conduct screenings from outside the chamber. This flexibility ensures the system's broad applicability across diverse microbial discovery campaigns. In this study, we focused exclusively on identifying antimicrobial activity from single environmental isolates against the target pathogen. However, higher‐order microbial interactions such as multi‐species synergy, induction, or cross‐feeding [45, 46] may be explored in future work, leveraging the Polychip to investigate novel antimicrobial mechanisms. Beyond antimicrobial discovery, this platform enables applications in microbiome research, synthetic biology screening, gut microbiome analysis, biomarker screening, and biotechnological discovery of novel compounds, microorganisms, and enzymatic activities. Its high efficiency and automated single‐cell resolution screening capability allow precise investigation of complex microbial interactions with enhanced throughput, supporting discovery campaigns and advancing drug discovery and consortia screening.

## 4 Method

### 4.1 Extraction of Environmental Bacterial Cells From Soil With High Viability

A direct cell extraction methodology from soil [28] was used to extract environmental microorganisms from soil. The soil sample was obtained from a pristine area (GPS: 34.979114, ‐97.521012, Purcell, Oklahoma, USA), and cells were immediately extracted after acquisition. Eight grams of fresh soil were cleaned to exclude wood, pebbles, and leaves before extraction. First, eight grams of soil were blended with 160 mL of 0.5% (v/v) Tween 20 solution (Sigma–Aldrich, MO, USA) using a high‐speed blender (Waring Laboratory Blenders, Conair, PA, USA) at 22 000 rpm for three cycles of 1 min each, with 1 min incubations on ice between cycles to prevent overheating. Second, 20 mL of blended soil slurry was carefully layered onto 18 mL of 80% (w/v) Nycodenz (1.42 g/mL) in a 50 mL centrifuge tube and centrifuged at 15 000×*g* for 40 min at 4°C with slow acceleration and deceleration. Third, the top two layers containing the extracted cells were transferred into a sterile 50 mL centrifuge tube and mixed with phosphate‐buffered saline (PBS) to a final volume of 35 mL. Fourth, the mixture was filtered through a 30 µm cell strainer to remove debris, then centrifuged at 15 000×*g* for 15 min at 4°C with slow acceleration and deceleration. Finally, the supernatant was discarded, and the cell pellet was resuspended in 5 mL of PBS buffer.

### 4.2 Device Fabrication

The droplet microfluidic device was fabricated in polydimethylsiloxane (PDMS, Sylgard 184 Dow Corning, MI, USA) using a conventional soft‐lithography method. The master molds for the PDMS microfluidic devices were fabricated using a standard photolithography process (SU‐8 3010, MicroChem, Westborough, MA, USA). The PDMS layers were then replicated from this master mold by mixing Sylgard 184 base and curing agent at a 10:1 ratio, mixing vigorously for 10 min, pouring onto the master molds in petri dishes, degassing for 10 min in a desiccator to remove air bubbles, and baking at 70°C for 12 h. The detailed fabrication protocol can be found in the supporting supplementary document.

### 4.3 Droplet Microfluidics Operation

All droplet transitions within each droplet manipulation step were induced by oil flow and buoyancy, providing the momentum to move droplets from the bottom layer (droplet generator) to the top layer (droplet sorter) of the Polychip. First, water‐in‐oil emulsion droplets with a diameter of 50 µm were generated using a cross‐junction droplet generator at the bottom layer of the Polychip using R2A as the aqueous phase containing environmental microorganisms and carrier fluorinated oil (Novec 7500, 3 M, MN, USA) containing 2% (wt/wt) surfactant (Pico‐Surf, Sphere Fluidics, Cambridge, UK). Second, the droplets containing environmental microorganisms were directed to a cylindrical droplet cultivation chamber situated above the droplet generator, where they were incubated for 48 h. Third, the cultivated droplets were transferred to the merging zone, located in the upper layer of the droplet cultivation chamber, where they were merged with droplets containing target pathogen cells. Fourth, the merged droplets were moved into a multiplex droplet cultivation chamber located in the upper layer of the droplet merger and incubated for another 48 h for co‐cultivation. Finally, the cultivated droplets were transferred, detected, and sorted, and only the “hit” droplets were dispensed onto agar plates at single‐droplet resolution for further analysis. Additional details are provided in the supporting supplementary document.

### 4.4 Graphical User Interface

A GUI was implemented using LabView (NI, Austin, TX, USA) to command up to three syringe pumps (Legato 101 dual infusion syringe pump, Holliston, MA, USA), an FPGA board (PCIe‐7842, National Instrument, Austin, TX, USA) connected with a terminal block (SCB‐68, National Instrument, Austin, TX, USA), and an Arduino microcontroller (Uno Rev3, Arduino, Monza, Italy). The GUI comprises three modules to control the aforementioned parts (Figures S5–S9) to conduct every droplet manipulation step with minimum human intervention. The human intervention steps are: (1) loading reagent syringes onto the syringe pumps, (2) flowing the interior of the tubing and device with oil (priming), (3) configuring operational parameters including flow rates and frequency/voltage of applied electric fields, and (4) monitoring the efficiency of droplet manipulation every six hours and adjusting the parameters (only if necessary). This approach minimized user‐induced errors, even when carrying out all six sequential droplet manipulation steps in a single chip. Additional information is detailed in the supporting supplementary document.

### 4.5 Inhibition Assay

We adopted two phenotype confirmation methodologies consisting of Kirby‐Bauer test and plate reader assay. All phenotypic analyses were performed in triplicates.

#### 4.5.1 Kirby‐Bauer Test

A pure R2A agar plate with a thin layer of target pathogen was used for the Kirby‐Bauer test. To prepare the pathogen layer, the target pathogen and “hit” strains were cultured in R2A at 30°C for 24 h and diluted to have an optical density of 0.4 (OD_600_). The pathogen layer was applied using a sterile cotton swab (Puritan Hospital Standard Cotton Swab, Hisco, Houston, TX, USA), evenly spread onto the agar surface, followed by a 1 h drying period. A sterile paper disk (Blank Paper Disk for AST, Hardy Diagnostics, Santa Maria, CA, USA) was placed on the plate, and 18 µL of the “hit” culture supernatant was pipetted onto the disk. Co‐cultivation on the agar plates was carried out at 22°C for 48 h before observing the results.

#### 4.5.2 Plate Reader Assay

Two different plate readers — Bioscreen C Pro (Molecular Devices LLC., San Jose, CA, USA) for OD_600_ measurement and BioTek Synergy H1 (Santa Clara, CA, USA) for GFP measurement — were used in the inhibition assay to evaluate the antimicrobial activities of live “hit” cells as well as their supernatants against the target pathogen. For cell preparation, “hit” strains and target pathogens were cultured in R2A broth at 25°C for 48 h in a shaking incubator and diluted to an OD_600_ of 0.15. For supernatant preparation, “hit” strains were cultured in R2A at 25°C for 72 h, then centrifuged (8000 rpm, 5 min), and the supernatant filtered through a 0.22 µm syringe filter (Tisch Scientific PTFE SYRINGE FILTERS 0.22 UM, Tisch Scientific, Cleves, Ohio, USA) to remove cells. A 96‐well plate (Cell Culture‐Treated, Flat‐Bottom Microplate, Fisherbrand, Waltham, MA, USA) was used, with each well containing 200 µL of a 1:1 mixture of the target pathogen and either live “hit” cells or supernatant. GFP fluorescence was used to quantify the antimicrobial activity against the pathogen, as “hit” cell growth could influence OD_600_ readings even if pathogen growth is inhibited. Conversely, OD_600_ measurements were employed to evaluate the antimicrobial activities of the supernatants, as only pathogen growth was considered. Both measurements were taken every three hours.

### 4.6 Gene‐Level Analysis

#### 4.6.1 DNA Extraction for Full‐Length 16S rRNA Sequencing and Whole Genome Sequencing

“Hit” cells were cultured in R2A at 25°C for 48 h in a shaking incubator for cell preparation. DNA extraction was performed using the E.Z.N.A. Bacterial DNA Kit (Omega Bio‐tek, GA, USA) following the manufacturer's protocol. For sequencing, full‐length 16S rRNA sequencing was conducted by ACGT (ACGT, IL, USA), while whole‐genome sequencing was performed by Plasmidsaurus (Plasmidsaurus, CA, USA).

#### 4.6.2 Phylogenetic Tree

To construct a phylogenetic tree depicting the genomic diversity of 79 “hits,” 16S rRNA sequencing results were trimmed to a uniform length of 755 base pairs. The trimmed sequences were aligned using MEGA11 [47] with the following parameters: 1) alignment algorithm: MUSCLE, 2) gap open/extend penalties: ‐400/0, and 3) clustering method/minimum diagonal length: UPGMA/24. Following sequence alignment, the phylogenetic tree was generated using iTOL [48].

#### 4.6.3 AntiSMASH

AntiSMASH [30] was conducted using the genebank data generated by whole genome sequencing for the top three “hits,” which showed prevalent antimicrobial activities against the target pathogens.

#### 4.6.4 LC‐MS

LC‐MS analysis was conducted at the Chemistry Mass Spectrometry Facility at Texas A&M University. For supernatant preparation, “hit” cells were cultured in T24 flasks (Surface Treated Sterile Tissue Culture Flasks, Vented Cap, Fisherbrand, Waltham, MA, USA) for 48 h at 30°C in a shaking incubator, then centrifuged. The supernatant was filtered through a 0.22 µm syringe filter, and 8 mL of the filtered supernatant was acidified with 50 mL of 10 N HCl. The supernatant was then extracted twice with 8 mL of ethyl acetate (1:1 extraction) [36]. The top aqueous layer was collected, dried in 1 mL aliquots on a hot block (Digital Cooling Drybath, Thermo Scientific, Waltham, MA, USA), and resuspended in 50 mL of methanol. The resulting compounds were injected, separated, and analyzed using gradient chromatography.

### 4.7 Statistical Analysis

Data are presented as mean standard deviation with the sample size of n = 3. Statistical comparison of various groups was analyzed using Student's t‐test (Prism; GraphPad).

## Conflicts of Interest

The authors declare no conflicts of interest.

## Supporting information




**Supporting File**: advs76745‐sup‐0001‐SuppMat.docx.

## Data Availability

The data that support the findings of this study are available in the supplementary material of this article.
