# Supplementary material for: Polychip‐A High‐Throughput Droplet Microfluidics Platform for Interrogating Microbial Interactions
Source: Adv Sci (Weinh). 2026 Jul 27;13(58):e23854. doi: 10.1002/advs.202523854 (PMC13403381; doi:10.1002/advs.202523854)
Supplement: Supplementary file 1 [file ADVS-13-e23854-s001.docx]

POLYCHIP - A HIGH-THROUGHPUT DROPLET MICROFLUIDICS PLATFORM FOR INTERROGATING MICROBIAL INTERACTIONS

Jeong Jae Han^+^, Adrian Ryan Guzman^+^, Aifen Zhou, Han Zhang, Rohit Gupte, Haemin Jung, Kaylee Delgado, Sini Skariah, Ali Sultan, Arul Jayaraman, Paul de Figueiredo*, and Arum Han*

J.J. Han, A. Han

Department of Multidisciplinary Engineering, Texas A&M University, College Station, USA

A. R. Guzman, H. Zhang, H. Jung, A. Han

Department of Electrical and Computer Engineering, Texas A&M University, College Station, USA

A. Zhou, A. Jayaraman, A. Han

Department of Chemical Engineering, Texas A&M University, College Station, USA

R. Gupte, A. Jayaraman, A. Han

Department of Biomedical Engineering, Texas A&M University, College Station, USA

K. Delgado

Department of Microbial Pathogenesis and Immunology, Texas A&M University, College Station, USA

S. Skariah, A. Sultan

Department of Microbiology and Immunology, Weill Cornell Medicine – Qatar, Cornell University, Qatar Foundation – Education City, Doha, Qatar

P. D. Figueiredo

Christopher S. Bond Life Sciences Center, University of Missouri, Columbia, USA

Department of Molecular Microbiology & Immunology, University of Missouri School of Medicine, Columbia, USA

Department of Veterinary Pathobiology, University of Missouri, Columbia, USA

Department of Chemical and Biomedical Engineering, University of Missouri, Columbia, USA

E-mail:

Arum Han, [arum.han@ece.tamu.edu](mailto:arum.han@ece.tamu.edu), Paul de Figueiredo, [PaulLifeScience@missouri.edu](mailto:PaulLifeScience@missouri.edu)

^+^ These authors contributed equally to this work.

^*^ Arum Han and Paul de Figueiredo are the co-corresponding authors.

1. Device Fabrication

The Polychip droplet microfluidics platform primarily comprises a droplet generation module for single-cell encapsulation (**Figure 1b** step1), a cylindrical in-drop cultivation chamber for culturing environmental microorganisms (**Figure 1b** step2), a droplet merging module for coalescing two different droplets (**Figure 1b** step3), a cylindrical multiplex chamber for sequentially processing multiple batches of droplets (**Figure 1b** step4), a droplet sorting module for selecting desired droplets (**Figure 1b** step5), and a basket-shaped “hit” chamber ^[1]^ for collecting and visualizing sorted droplets. Each droplet module, except for the cylindrical chambers, consists of three layers: a bottom base layer (2 mm thickness) for channel sealing, a middle microfluidic channel layer (100 µm thickness) for droplet processing, and a top microvalve layer (2 mm thickness) for controlling flow and droplet propagation. Therefore, in total, the vertically stacked Polychip is composed of 14 layers, 12 layers for the droplet generation module, droplet merging module, droplet sorting module, and basket-shaped “hit” chamber, and then 2 layers for the cylindrical chambers.

All layers were fabricated in polydimethylsiloxane (PDMS) using conventional soft lithography methodologies ^[2-5]^. The fabrication step of each layer is elaborated below. The height of all microfluidic channels is 50 µm.

1.1. Base Layer

The base layer does not consist of any microstructures, hence, a mastermold is not required for this layer. Thus, PDMS was poured in a petri dish, degassed, and cured to obtain a 2 mm thick base layer.

1.2. Microfluidic Layer

The microfluidic layer is a set of layers containing droplet manipulating microstructures, such as a droplet generator, droplet merger, and droplet sorter. All designs utilized a sloped droplet transition structure for highly efficient droplet manipulation, namely curved transition ^[6]^. Thus, mastermolds for all designs were fabricated using a two-photon lithography tool (Nanoscribe Photonics GT2, Nano Scribe, Karlsruhe, Germany) as shown in our previous work ^[6-8]^. To obtain a 100 µm thick PDMS layer, a spin coater (WS 400B NPP LITE, Laurell Technologies Corporation, Lansdale, PA, USA) was used at 3,500 RPM for 35 s. Detailed specifications of each droplet-manipulating microfluidic channel are described below.

1.2.1. Droplet Generator

A three-inlet flow focusing droplet generator design ^[9]^ was adopted, which consists of three inlet channels each being 35 µm wide for oil and aqueous flow.

1.2.2. Droplet Merger

An interdigitated electrode (IDE) droplet merger design, which we have reported previously ^[4]^, was adopted. The channel width is 70 µm, and the IDE design has 10 µm wide 475 µm long electrode fingers, with finger-to-finger distance of 10 µm. The electrodes are covered by an 800 nm thick Si_3_N_4_ insulation layer.

1.2.3. Droplet Sorter

A dielectrophoretic-based droplet sorter design ^[10]^ was adopted. The shielding channel width is 250 µm, filled with liquid metal ^[11]^. The channel widths of the main, “hit” droplet, and waste channels are 250 µm, 130 µm, and 140 µm, respectively.

1.3. Microvalve Layer

The microvalve layer is a 2 mm thick PDMS layer embedding a 50 µm deep micro-channel structures to pressurize or release the thin microfluidic channel (microfluidic layer), which enables actuating the microvalve. Mastermolds for the microstructures of the microvalve were fabricated by a conventional photolithography methodology ^[2-5]^. To obtain the 2 mm thick PDMS block, the same methodology described above in the *Base layer* was used. Two different shapes of valve designs were used, which were square-shaped valves (50 µm × 50 µm) to control oil/aqueous flow and oval-shaped valves (2 mm in diameter) to control the cylindrical chamber. Detailed fabrication methods were adopted from our previous work ^[6]^.

1.4. Cylindrical Chamber Layer

The cylindrical chamber layers, the in-drop cultivation chamber, and the multiplex chambers were fabricated using polycarbonate (PC) molds using a CNC milling machine (MDX-50 benchtop CNC, Roland, Irvine, CA, USA). The milled PC master mold resulted in a final chamber height of 10 mm and diameter of 2 mm. Detailed fabrication methods were adopted from our previous work ^[12]^.

Plasma treatment using oxygen plasma (Harrick Plasma PDC-001-HP, 18 W for 120 s) was conducted to bond each PDMS part, with details of the bonding procedures described below.

1.4.1. Valve Layer Bonding to the Microfluidic Layer

Alignment markers were incorporated into each layer of the fluidic channels and the 3D milled structures to accomplish iterative stacking of adjacent layers.

1.4.2. Merging/Sorting Module Bonding to Cylindrical Chambers

High-profile cylindrical chambers pose alignment challenges due to focusing issues during microscopic observation. To address these difficulties, a physical alignment block was embossed on the top of each cylindrical chamber layer. Corresponding extruded blocks were added to the bottom of each merging and sorting module, precisely scaled to match the embossed features. As a result, the embossed and extruded alignment features fit together seamlessly during module and chamber assembly, enabling precise and simplified alignment.

2. Droplet Manipulation

Droplet manipulation at each step was primarily driven by three forces: pump-driven aqueous flow, buoyancy, and dielectrophoresis (DEP). Droplet movement was facilitated by the external aqueous pressure of carrier fluorinated oil (Novec 7500, 3M) containing 2% (wt/wt) surfactant (Pico-Surf™, Sphere Fluidics, Cambridge, UK), enabling droplets to flow through the microfluidic channels. Droplet buoyancy within the continuous phase caused droplets to float up, controlling droplet sequencing in a first-in-first-out (FIFO) manner within the droplet cultivation chamber, controlling transitions between each droplet manipulation step. Lastly, DEP was used to weaken droplet surface tension for merging or redirecting droplet trajectory during sorting.

2.1. Single-Cell Encapsulation into Droplets

Single-cell droplet encapsulation was performed (**Figure 1b**, step 1). Environmental microorganisms extracted and suspended in R2A medium were concentrated to an optical density (OD) of 0.2 measured by a spectrophotometer (NanoDrop, Thermo Scientific™, Invitrogen™, PA), and then diluted 100-fold in R2A to achieve single-cell encapsulation for each droplet based on Poisson’s distribution. A flow-focusing droplet generator ^[3]^ with channel width of 35 µm and height of 50 µm was used to encapsulate single bacterial cells in droplets having a droplet diameter of 50 µm. Approximately 400 µL of environmental microorganisms in droplets (2.18×10^6^ droplets) were generated, which were then cultivated in the vertical droplet cultivation chambers. For this step, flowrates of 400 µL/h and 300 µL/h were used for oil and cell-in-media flow, respectively.

2.2. In-drop Cultivation

In-drop cultivation was conducted as described (**Figure 1b**, step 2). Droplets encapsulating each extracted environmental microbe were introduced through the bottom sloped inlet of the vertical cylindrical droplet cultivation chamber (2 mm diameter, 10 mm height) filled with oil for incubation. Due to buoyancy, droplets entering the chamber first floated up to the top of the chamber, while later droplets settled beneath the earlier droplets. This allowed first-in first-out of droplets when they exit the vertical droplet incubation chamber from the top outlet. In-droplet cultivation was carried out for 48 h at 22°C before exiting the incubation chamber for further droplet assays. Growth analysis of the environmental microbes was conducted in advance (**Figure S1**) to determine the cultivation time sufficient for broad ranges of environmental microbes to reach stationary growth phase. Based on this, 48 h cultivation time for in-drop cultivation was used.

**Figure S1.** Growth curves of four environmental samples cultured in a 96 well-plate with R2A as the culture medium. All four environmental samples tested reached stationary phase within approximately 30 h. R2A media only was used as blank control. Data are presented as the mean ± SD (n = 3).

2.3. Droplet Merging

Droplet merging was conducted as shown in **Figure 1b**, step 3. Droplet merging entailed combining droplet contents from in-drop cultured environmental microbes (cultivated for 48 h) with droplets containing 7–10 target pathogen cells expressing GFP, enabling co-cultivation to observe antimicrobial activities of cultivated environmental microbial strains. The droplet merging step was similar to what we reported previously ^[4]^, with the merged droplets forming a final droplet diameter of ~90 µm. An in-droplet cultivation test of GFP-expressing *Acinetobacter baumannii* was conducted (**Figure S2**) to assess the GFP signal intensity of negative control (i.e., normal growth where co-cultivated environmental microbial strains show no antimicrobial activities). For this step, flowrates of 20 µL/h, 400 µL/h, and 100 µL/h were used for droplet reflow, oil flow, and pathogen-in-media flow, respectively. To generate an electric field (EF) from the IDE pattern for droplet merging, a continuous sinusoidal signal of 8.5 kHz and 250 V was applied.

**Figure S2.** Time course GFP channel fluorescent microscopy images of GFP-*A. baumannii* growth in droplets.

2.4. Co-Cultivation with Pathogen Cells in Droplets

Co-cultivation with pathogen cells in droplets was conducted in the cylindrical multiplex droplet cultivation chamber (**Figure 1b**, step 4). Co-cultivation of extracted environmental microbes and target pathogen cells expressing GFP was performed at 22°C in the multiplexed droplet cultivation chamber, enabling back-to-back screening of large batches of environmental microbial library. As shown in **Figure S3**, the multiplexed chamber consist of two vertically arranged chambers for droplet cultivation and eight microvalves (V1 and 5: cultured droplet release, V2 and 6: oil release, V3 and 7: droplet inlet, V4 and 8: oil infusion) to direct droplet flow and oil release. Detailed roles of the valves are described below.

*2.4.1. Valves V3 and V7*

Control inlets for the merged droplets from the droplet merging module.

*2.4.2. Valves V2 and V6*

Control outlets for releasing carrier oil introduced with merged droplet reflow from the droplet merging module.

*2.4.3. Valves V1 and V5*

Control outlets for releasing incubated droplets, connected to the droplet sorting module.

*2.4.4. Valves V4 and V8*

Control inlets for oil used to push the incubated droplets through valves V1 and V5.

The aforementioned eight valves (V1-8) were opened and closed to fill and release droplets into and from the cylindrical multiplexing chambers. The detailed operation of valves to fill and release the chambers with droplets are described below.

- *Step 1*: Store the merged droplets only in *Chamber 1*. Open valves V2 and V3 of *Chamber 1* to release the carrier oil and introduce the merged droplets, respectively. All other valves in both *Chamber 1* and *Chamber 2* remain closed during this step.
- *Step 2*: Transfer the incubated droplets from Chamber 1 to the sorting module while Chamber 2 stores newly merged droplets from the merging module. In *Chamber 1,* open valves V1 and V4, and close V2 and V3. Oil introduced through V4 pushes the incubated droplets, which are released through V1 to the sorting module. In *Chamber 2*, open valves V6 and V7, and close V5 and V8, so that V6 releases carrier oil and V7 introduces the merged droplets.
- *Step 3*: Transfer the incubated droplets from *Chamber 2* to the sorting module while *Chamber 1* stores newly merged droplets. In *Chamber 1*, open V2 and V3, and close V1 and V4 to release carrier oil and introduce the merged droplets, respectively. *Chamber 2* maintains the same valve configuration as in *Step 2* to continue emptying.
- *Iteration*: Repeat *Steps 2* and *3* to enable multiplexed incubation of various droplet batches.

This allowed for seamless storing and releasing of droplets without impacting adjacent chambers, allowing for the expansion of multiplexing (i.e., 4x4, 5x5, and 6x6 chambers).

**Figure S3.** Illustrations of the multiplexed droplet cultivation chambers controlled by eight microvalves to store and release cultivated droplets towards the droplet sorting layer.

2.5. Droplet Sorting

Droplet sorting was conducted as shown in **Figure 1b**, step **5**. Droplet fluorescence detection was performed using a laser-based fluorescence detection system. A 470 nm laser (LRS-0473-PFM-00100-03, Laserglow Technologies, Toronto, Ontario, Canada) was used to excite the merged droplets, and the GFP signal intensity from each droplet (**Figure S2-3**) was measured by photomultiplier tubes (H10721-110, Hamamatsu Photonics, Shizuoka, Japan). Based on the GFP intensity, DEP force was applied to the target droplets to redirect the “hit” droplet trajectory to the “hit” outlet ^[10]^. Sorted droplets were then stored in the “hit” droplet chamber and subsequently dispensed onto agar plates for downstream assays. For this step, flowrates of 50 µL/h, 200 µL/h, and 80 µL/h were used for droplet reflow, spacing oil flow, and bias-oil flow, respectively. To generate EF from electrodes to redirect droplets for sorting, a trigger signal of 9 kHz and 1,400 V was employed, which was generated by a function generator (DG4102, Rigol Technologies, Beijing, China) and a high-voltage amplifier (TREK-2220, Trek Technologies, Portland, OR, USA).

3. Automated Droplet Sorting Mechanism

The automated droplet sorting system consists of an optical detection system, a LabVIEW-based field programmable gate array (FPGA) graphical user interface (GUI), and a droplet microfluidic sorting function. The optical detection system and droplet sorting functionality are detailed in our previous work ^[1, 7]^. To selectively sort “hit” droplets exhibiting weak or negligible GFP signal, a droplet sorting algorithm was developed to guide the user to adaptively adjust the sorting threshold during droplet sorting, taking into account variations in min/max and a dynamic signal-to-noise ratio (SNR) of the fluorescence signal. This resulted in the sorting of less than 1% of the entire input library population as of “hits” for downstream analysis.

**Figure S4** illustrates the results of in-droplet cultivation of multidrug-resistant (MDR) *Pseudomonas aeruginosa*, highlighting differences in GFP intensities at various time points and demonstrating the droplet sorting function achieving > 99% sorting efficiency based on GFP intensity. In-drop growth analysis of the pathogen was performed to establish a baseline and maximum thresholds for droplet sorting optimization by observing the lowest and highest GFP intensities of droplets containing only pathogen cells or empty droplets (**Figure S4a, b**). **Figure S4a** depicts GFP intensities of droplets containing MDR *P. aeruginosa* with (black line) and without (red line) antibiotic kanamycin, respectively. An example of GFP channel microscopic images at 0 h and 8 h are shown in **Figure S4b**. **Figure S4c** shows droplet GFP intensity as droplets pass through the laser-based fluorescence detection region. Using the established baseline and maximum thresholds, the adaptive algorithm (see below in detail) was used to determine the sorting threshold (orange line) to selectively isolate droplets exhibiting a very weak GFP signal. Here, the configured threshold was inversely related to the expected biological inhibition strength of the sorted droplets. Specifically, setting a lower threshold results in the selection of only those droplets with weaker or no fluorescence intensity relative to the threshold, thereby isolating candidates with potentially higher inhibitory activity.

**Figure S4d** presents a flowchart illustrating the algorithm's adaptive threshold process, with a detailed explanation of the algorithm below.

1. The initial threshold is set by the user.
2. If a droplet expresses lower GFP intensity than this set threshold, then the droplet is sorted. The number of sorted droplets and waste droplets are counted.
3. If the ratio of the number of sorted droplets over the sum of sorted and abandoned droplets is over 1%, then the threshold is decreased by 5% of the adaptive value and vice versa.
4. Repeat steps 2-3.

**Figure S4.** Workflow of the droplet sorting function. **a:** GFP intensities of droplets containing GFP-expressing MDR *P. aeruginosa* with (black line) and without (red line) 50 µL of antibiotic kanamycin. **b:** GFP channel microscopic images of droplets containing GFP-expressing MDR *P. aeruginosa*. Images include pathogen droplets in pure R2A at 0 h, pathogen droplets cultivated in R2A at 8 h, and pathogen droplets with 50 µL of kanamycin at 8 h. **c:** Screenshot of the GUI depicting the measured GFP intensities of droplets passing through the laser excitation spot. The orange line indicates the threshold used to determine droplet sorting, where droplets with GFP intensity below this threshold are sorted, while those above the threshold are discarded. **d**: A flowchart depicting the algorithm used to adaptively sort less than 1% of droplets exhibiting negligible GFP intensity, representing “hits” exhibiting antimicrobial activities. Data are presented as the mean ± SD (n = 100). The statistical significance between different groups was analyzed using Student’s t-test (Prism; GraphPad).

4. GUI of the Polychip Platform

The GUI of the Polychip platform comprises of three modules – a Syringe Pump Controller Module, a Droplet Sorting & Merging Module, and a Data Recording Module (**Figure S5**). The Syringe Pump Controller Module controls up to three individual syringe pumps and sets parameters for the size of syringes, flowrates, and target volume. The Droplet Sorting & Merging Module is used to set the related parameters for droplet sorting (threshold, DEP frequency, DEP actuation duration, and delay) and merging (DEP frequency and output voltage). Additionally, the sensitivity of the PMT sensors, waveform signal generation for droplet sorting and merging, and threshold for real-time signal processing from the PMT sensors are all controlled by subfunctions in the GUI algorithm. Finally, the Data Recording Module is used to save experimental data such as PMT signal, threshold settings, and counts based on triggering (on/off) of the sorting signal as time elapses during the experimental operation.

**Figure S5.** The LabVIEW GUI providing control, signal acquisition, and data processing for the Polychip platform. The yellow highlighted area is the Syringe Pump Controller Module used to set the durations of input fluid flow, flow rates, and volume. The red highlighted area is the Droplet Sorting & Merging Module for configuring detection sensitivity, trigger parameters, and monitoring real-time GFP intensity detection. The blue highlighted area is the Data Recording Module for saving system parameters, settings, and results from sorting.

4.1. GUI Setup

To operate the GUI for each droplet assay, a user is required to configure several parameters for communication setup, output file path setup, Arduino initialization, and syringe pump setup. The step-by-step instructions with corresponding program captures (**Figures S6 – 9**) for the setup are written below.

- Setting up communication properties between a laptop and hardware components
  - Select the corresponding COM port number for the syringe system (reference: Device Manager).
  - Select the corresponding COM port number for the Arduino (reference: device manager)

**Figure S6.** Communication module used to configure COM ports for the syringe pumps and Arduino.

- Setting up the output file path and file name
  - Output file paths should be created manually before program execution.

**Figure S7.** Data recording module to define output file name and set the output file path.

- Executing and initializing the program
  - Click the “🡺” button at the top left of the LabView window.
    - If you encounter any error at this stage, confirm whether the communication setup and data recording configuration were processed correctly.
  - Initialize Arduino
    - Click the “Initializer” button.
    - Enter the desired values of each parameter.
    - Click the button for each parameter to setup the parameter.
    - NOTE: For droplet merging only, the toggle button should be activated to provide a continuous sinusoidal signal from the system.

**Figure S8.** Droplet sorting and merging module to configure and set parameters related to the output signal from the Arduino.

- Configuring syringe pump parameters
  - Select and input desired parameter values into the parameter boxes.
  - Click the corresponding “Set pump” button to finalize the parameters for each syringe pump.

**Figure S9.** Syringe pump module to configure parameters related to the syringe pumps.

- Closing and finalizing the program
  - Click the STOP button in the communication module (see **Figure S6**).

5. Droplet Dispensing

An automated in-house droplet dispenser ^[13]^ was used for dispensing “hit” droplets onto agar plates (**Figure S10**). Droplets were dispensed at single-droplet resolution onto the agar plate in an ordered grid pattern to prevent overlap of single colonies, resulting in single colony formation where “hit” droplets were dispensed.

**Figure S10.** Illustration of the droplet dispensing pipeline.

6. Efficiency of the Polychip Screen

Efficiency was determined by the combined efficiency of the microfluidic droplet manipulation assays consisting of droplet generation, droplet merging, and droplet sorting. The Polychip integrates a reliable flow-focusing droplet generator ^[9]^, an IDE droplet merger ^[4]^, and a linear droplet sorter ^[7]^. To ensure an ideal microfluidic environment and secure near-perfect efficiency for each individual droplet manipulation step, the platform was operated at a lower throughput relative to the maximum possible throughput of each assay. The overall efficiency of the Polychip was then calculated by Equation 1, multiplying the individual accuracy of these sequential steps, as detailed in Table S1.

**Table S1.** Efficiency of each droplet manipulation step and the overall efficiency of the Polychip-based droplet manipulation.

|  | Droplet Generation | Droplet Merging | Droplet Sorting | Polychip |
| --- | --- | --- | --- | --- |
| Efficiency | 100% | 100% | 99.78 ± 0.19% | 99.7% |

$\boldsymbol{Efficiency}_{\boldsymbol{Polychip}}\boldsymbol{=\Pi(Efficiency of each droplet manipulation assay)}$	*(1)*

$$\boldsymbol{Efficiency}_{\boldsymbol{Polychip}}\boldsymbol{=\Pi(\ldots}$$

$$\boldsymbol{Efficiency}\left( \boldsymbol{Droplet Generation} \right)\boldsymbol{, \ldots}$$

$$\boldsymbol{Efficiency}\left( \boldsymbol{Droplet Merging} \right)\boldsymbol{, and\ldots}$$

$$\boldsymbol{Efficiency}\left( \boldsymbol{Droplet Sorting} \right)\boldsymbol{)}$$

$\boldsymbol{Efficiency}_{\boldsymbol{Polychip}}\boldsymbol{=\Pi}(100\%, 100\%, 99$*.*78 *±* 0.19%)

$\boldsymbol{Efficiency}_{\boldsymbol{Polychip}}\boldsymbol{=}99.78$ *±* 0.19% ≈ 99.7%

Here, the efficiency defined in Table S1 represents the success rate of each individual microfluidic assay under optimal mechanical conditions, excluding biological efficiency. Specifically, the “99.78 ± 0.19% sorting efficiency” refers to the mechanical routing accuracy, which is the system's success rate in physically routing a droplet once a trigger signal is generated. In contrast, our selection stringency is defined by a threshold targeting droplets with absent or significantly reduced GFP intensities, specifically those ranked in the bottom of the population, as demonstrated in Figure S4. Consequently, while the system is mechanically accurate, our stringent thresholding means a significant number of “waste” droplets may still contain antimicrobial “hits” but did not meet this rigorous selection criteria.

Regarding the biological efficiency of the Polychip screen, hit recovery efficiency can vary significantly, depending on both target pathogens and the diversity of microbial library input for the screening. Environmental factors including season and location influence the microbial diversity ^[14]^. In addition, we could only confirm hits that were culturable in both solid (agar) and liquid (broth) media. Consequently, calculating absolute recovery efficiency remains challenging.

Regarding the mechanical longevity of the Polychip, we implemented an adaptive thresholding algorithm (Figure S4) and utilized an anti-vibration optical table to isolate mechanical interference (vibrations or external forces). This ensures that the relationship between the chosen threshold and biological inhibition strength remains robust, even as experimental parameters fluctuate. In terms of microfluidic longevity, our previous studies have established that multi-day microfluidic operations are feasible, provided the channels remain free of large particulates that cause clogging ^[1, 4, 15]^. To demonstrate the stability of droplets during in-drop incubation within the Polychip, we encapsulated *E. coli* (K12) in R2A medium into droplets (diameter: 140 µm) and incubated them for 48 h at 37°C. As observed in Figure S11, the droplets remained stable following the incubation period.

**Figure S11.** Microscopic images demonstrating droplet stability over 48 h of incubation (37°C). Left: 0 h; right: 48 h of incubation.

7. Processing Time of Each Droplet Assay Step

To ensure that each droplet received a near-identical in-drop incubation period, which is particularly critical during the co-cultivation stage of phenotypic inhibition analysis, we standardized the duration of both droplet merging and sorting to approximately 20 h. Consequently, the total in-droplet co-cultivation time for each droplet prior to sorting was 68 h (48 h initial co-cultivation + 20 h processing time of the droplet merging).

In here, droplets fill the cylindrical chamber as they leave the previous assay step. Consequently, addition-al filling time is not required. Also, the travel time of droplets from the bottom to the top of the cylindrical chamber via buoyancy is approximately 14 seconds. Therefore, the droplet processing time in the chamber is negligible. Thus, the expected residence times of each droplet are nearly identical if the droplet pro-cessing times of the previous and next assays are the same, following the first-in-first-out (FIFO) principle ^[12]^.

The cumulative incubation times for each step is illustrated in **Figure S12**.

**Figure S12.** An illustration showing the detailed timelines of each droplet assay.

8. Polychip Screening against MDR *Acinetobacter baumannii*

To demonstrate the reproducibility and reliability of the Polychip, we conducted Polychip screening against the MDR pathogen *A. baumannii*. As shown in **Table S2**, we encapsulated 3.14×10^6^ environmental microbes, obtained from a ranch area at the Texas A&M University (Texas, USA), into droplets and conducted the Polychip screen. This resulted in 2.77×10^4^ “hit” droplets. Off-chip validation revealed that 24 out of 78 (31%) tested “hits” exhibited strong antimicrobial activity against MDR *A. baumannii*. These results demonstrate that Polychip is a reproducible and reliable screening platform, even when different sets of input microbes and target pathogens are employed.

**Table S2.** Environmental microbial screening results against MDR *A. baumannii* through Polychip.

| Target Pathogen | Input Microbes | Generated Droplets | Hit Droplets | Hits with ZOI (%) |
| --- | --- | --- | --- | --- |
| *A. baumannii* | 3.14×10^6^ | 3.05×10^6^ | 2.77×10^4^ | 31%  (24 / 78 hits) |

**Figure S13.** Siderophore detection result from the supernatant of P1 and P10 using the CAS agar testing method.

**Figure S14.** Box-and-whisker plot demonstrating the reduction in antimicrobial activity of three high-priority “hits” (P1, P10, and P27) as the concentration of FeCl_3_ increases. N = 3.

**References**

[1]	H. S. Kim *et al.*, "High-throughput droplet microfluidics screening platform for selecting fast-growing and high lipid-producing microalgae from a mutant library," *Plant Direct,* vol. 1, no. 3, p. e00011, Sep 2017, doi: 10.1002/pld3.11.

[2]	J. A. Wippold, H. Wang, J. Tingling, J. L. Leibowitz, P. de Figueiredo, and A. Han, "PRESCIENT: platform for the rapid evaluation of antibody success using integrated microfluidics enabled technology," *Lab Chip,* vol. 20, no. 9, pp. 1628-1638, May 7 2020, doi: 10.1039/c9lc01165j.

[3]	A. Lashkaripour, C. Rodriguez, L. Ortiz, and D. Densmore, "Performance tuning of microfluidic flow-focusing droplet generators," *Lab Chip,* vol. 19, no. 6, pp. 1041-1053, Mar 13 2019, doi: 10.1039/C8LC01253A.

[4]	J. J. Han, H. Zhang, Y. Li, C. Huang, A. R. Guzman, and A. Han, "High-Efficiency Interdigitated Electrode-Based Droplet Merger for Enabling Error-Free Droplet Microfluidic Systems," *Anal Chem,* vol. 96, no. 34, pp. 13906-15, Aug 15 2024, doi: 10.1021/acs.analchem.4c02376.

[5]	A. R. Guzman, H. S. Kim, P. de Figueiredo, and A. Han, "A three-dimensional electrode for highly efficient electrocoalescence-based droplet merging," *Biomed Microdevices,* vol. 17, no. 2, p. 35, Apr 2015, doi: 10.1007/s10544-014-9921-x.

[6]	H. Zhang *et al.*, "An ultra high-efficiency droplet microfluidics platform using automatically synchronized droplet pairing and merging," *Lab Chip,* vol. 20, no. 21, pp. 3948-3959, Nov 7 2020, doi: 10.1039/d0lc00757a.

[7]	H. Zhang *et al.*, "NOVAsort for error-free droplet microfluidics," *Nat Commun,* vol. 15, no. 1, p. 9444, Nov 1 2024, doi: <https://doi.org/10.1038/s41467-024-52932-z>.

[8]	H. Zhang *et al.*, "FIDELITY: A quality control system for droplet microfluidics," *Sci Adv,* vol. 8, no. 27, p. eabc9108, Jul 8 2022, doi: 10.1126/sciadv.abc9108.

[9]	X. Chen, T. Glawdel, N. Cui, and C. L. Ren, "Model of droplet generation in flow focusing generators operating in the squeezing regime," *Microfluidics and Nanofluidics,* vol. 18, no. 5-6, pp. 1341-1353, 2014, doi: 10.1007/s10404-014-1533-5.

[10]	C. Huang, Y. Jiang, Y. Li, and H. Zhang, "Droplet Detection and Sorting System in Microfluidics: A Review," *Micromachines (Basel),* vol. 14, no. 1, Dec 30 2022, doi: <https://doi.org/10.3390/mi14010103>.

[11]	C. Koo, B. E. LeBlanc, M. Kelley, H. E. Fitzgerald, G. H. Huff, and A. Han, "Manipulating Liquid Metal Droplets in Microfluidic Channels With Minimized Skin Residues Toward Tunable RF Applications," *Journal of Microelectromechanical Systems,* vol. 24, no. 4, pp. 1069-1076, 2015, doi: 10.1109/jmems.2014.2381555.

[12]	J. Dai, H. S. Kim, A. R. Guzman, W.-B. Shim, and A. Han, "A large-scale on-chip droplet incubation chamber enables equal microbial culture time," *RSC Advances,* vol. 6, no. 25, pp. 20516-20519, 2016, doi: 10.1039/c5ra26505c.

[13]	H. Jung *et al.*, "Size-independent and automated single-colony-resolution microdroplet dispensing," *Lab Chip,* vol. 25, no. 23, pp. 6157-6169, Nov 18 2025, doi: 10.1039/d5lc00374a.

[14]	A. Pitcher, L. Villanueva, E. C. Hopmans, S. Schouten, G. J. Reichart, and J. S. Sinninghe Damste, "Niche segregation of ammonia-oxidizing archaea and anammox bacteria in the Arabian Sea oxygen minimum zone," *ISME J,* vol. 5, no. 12, pp. 1896-904, Dec 2011, doi: 10.1038/ismej.2011.60.

[15]	J. Dai *et al.*, "Microfluidic droplets with amended culture media cultivate a greater diversity of soil microorganisms," *Appl Environ Microbiol,* vol. 91, no. 3, p. e0179424, Mar 19 2025, doi: 10.1128/aem.01794-24.
